# Mucosa-associated lymphoid tissue lymphoma translocation 1 as a novel therapeutic target for rheumatoid arthritis

**DOI:** 10.1038/s41598-017-12349-9

**Published:** 2017-09-19

**Authors:** Chang Hoon Lee, Su Jeong Bae, Miok Kim

**Affiliations:** 1 Bio & Drug Discovery Division, Center for Drug Discovery Technology, Korea Research Institute of Chemical Technology (KRICT), Daejeon, Republic of Korea; 2 Immunotherapy Convergence Research Center, Korea Research Institute of Bioscience & Biotechnology (KRIBB), Daejeon, Republic of Korea

## Abstract

Emerging evidence suggests that mucosa-associated lymphoid tissue lymphoma translocation 1 (MALT1) is a key regulator of inflammatory diseases; however, the pathological role of MALT1 in rheumatoid arthritis (RA) is not well understood. Consequently, this protein has not been therapeutically targeted for the treatment of RA. MALT1 plays a role in the paracaspase pathway, has proteolytic activity and is involved in the regulation of inflammatory responses. In this study, we found that the MALT1-targeting inhibitory small molecule, MALT1 selective inhibitor 2-chloro-N-[4-[5-(3,4-dichlorophenyl)-3-(2-methoxyethoxy)-1H-1,2,4-triazol-1-yl]phenylacetamide (MI-2) strongly suppresses the differentiation of monocytes into osteoclasts in the absence or presence of the inflammatory cytokine tumour necrosis factor α. Furthermore, MI-2 ameliorates pathologic bone erosion and synovitis in an *in vivo* mouse model of collagen-induced arthritis. Mechanistically, MI-2 blocked expression of the master osteoclast regulator – nuclear factor of activated T cells 1 (NFATc1) – by inhibiting nuclear factor κB (NF-κB), which is a critical regulator of NFATc1. These findings highlight the important regulatory role of MALT1 in the NF-κB–NFATc1-signalling axis during osteoclastogenesis and suggest that targeting MALT1 is a promising treatment option for rheumatoid arthritis.

## Introduction

Rheumatoid arthritis (RA) is a chronic inflammatory disease that is characterised by joint destruction caused by infiltrating leukocytes, such as monocytes. RA is one of the most prevalent inflammatory diseases and affects up to 0.6% of the adult population, thereby representing a major public health burden^1^. Nonetheless, a limited number of therapeutic drugs are currently available for treating arthritis. Although several biological agents, such as tumour necrosis factor α (TNFα) blockers, display potent efficacy in alleviating RA symptoms, a considerable percentage of anti-TNFα-treated patients do not show a significant clinical response^2,3^. Thus, there remains an unmet need for therapeutic drugs that are based on novel mechanisms. The cause of RA is not clear; however, the aggravation of RA symptoms is currently thought to be caused by an increased number of osteoclasts in the joints of RA patients, which may be due to the differentiation of infiltrating monocytes^4,5^. An increase in osteoclasts mediates the destruction of cartilage and bone in the joints of RA patients, which frequently results in deformities and loss of joint function^6,7^. Therefore, blocking the differentiation of monocytes into osteoclasts could represent a novel effective therapeutic approach for RA. However, until recently, there were no known therapeutic reagents that could block this process.

Recently, mucosa-associated lymphoid tissue lymphoma translocation 1 (MALT1) has been shown to be a key modulator of immune cell function^8^. MALT1 is a part of the paracaspase pathway and has proteolytic activity^9,10^. Because many MALT1 substrates are involved in regulating inflammatory responses, the protease activity of MALT1 has emerged as an interesting therapeutic target. However, the role of MALT1 in RA has not been elucidated. In this study, the effects of a selective MALT1 inhibitor, on the differentiation of monocytes into osteoclasts was evaluated to assess the role of MALT1 in RA. MALT1 selective inhibitor 2-chloro-N-[4-[5-(3,4-dichlorophenyl)-3-(2-methoxyethoxy)-1H-1,2,4-triazol-1-yl]phenylacetamide (MI-2) directly binds to MALT1 and suppresses its protease function. MI-2 is non-toxic to mice and displays selective activity against ABC-DLBCL cell lines *in vitro* and xeno-transplanted ABC-DLBCL tumours *in vivo*. Those effects are thought to be caused by the irreversible inhibited cleavage of MALT1 substrates^11^. The findings of this study provide important insights into the potential of MALT1 as a novel therapeutic target and the applicability of MI-2 or other selective MALT1 inhibitors for treating RA.

## Results

### MI-2 inhibited osteoclast formation

**Figure 1:**
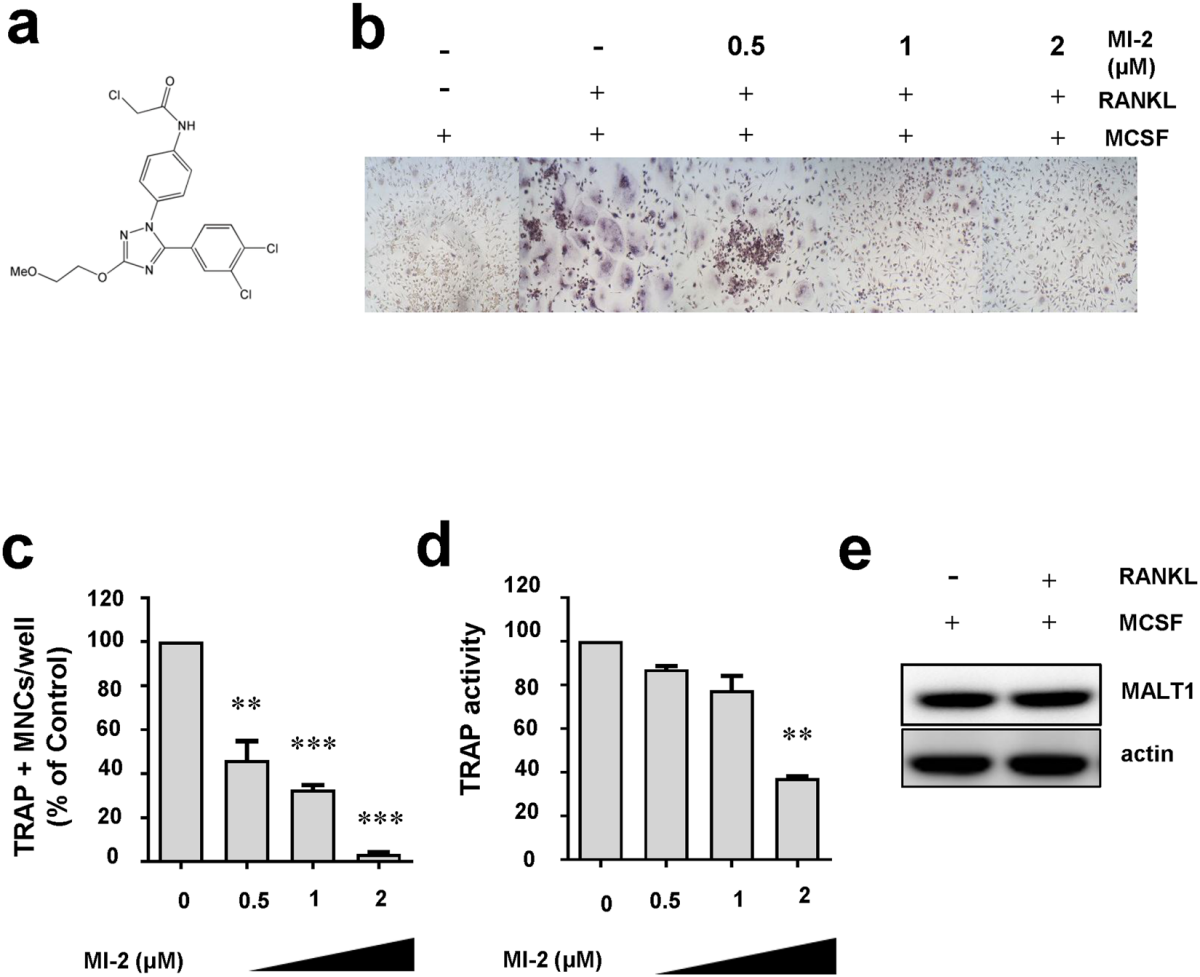
Inhibitory effects of the MALT1 inhibitor MI-2 on osteoclast formation. The inhibitory effects of MI-2 on osteoclast formation from monocytes was determined by performing *in vitro* osteoclast-differentiation assays. (**a**) Structure of the small molecule MI-2, a MALT1 inhibitor. (**b**) Representative images from more than 3 independent experiments are shown. (**c**) Relative value (%) of the number of TRAP-positive osteoclasts per well. Pooled results are shown from 3 independent experiments. (**d**) TRAP enzyme activities (%) in untreated cells and cells treated with the indicated concentrations of MI-2. (**e**) Human monocytes were incubated in differentiation medium (α-MEM containing 10% FBS, 20 ng/mL MCSF, and 50 ng/mL RANKL). Protein expression in the cell lysates was analysed by immunoblotting with an anti-MALT1 antibody. The level of β-actin was analysed as a loading control. Full-length blots are presented in Supplementary Figure 1. The results shown are representative of 3 different independent experiments. *, **, and *** indicate significant differences from control (untreated) cells based on 2-tailed unpaired Student’s *t*-tests at *P* < 0.05, *P* < 0.01, and *P* < 0.001, respectively. The error bars show standard error of the mean (s.e.m.) values.

Increased numbers of osteoclasts in the joints of patients with RA have been frequently reported^12^. This phenomenon results in enhanced osteoclast-dependent destruction of cartilage and bones in the joints, with subsequent deformity and loss of joint function. In this study, we investigated the role of MALT-1 in osteoclast formation by evaluating the effects of the MALT1 inhibitor MI-2 (Fig. 1a) on monocyte differentiation into osteoclasts in the presence of receptor activator of nuclear factor kappa-B ligand (RANKL) and macrophage colony-stimulating factor (MCSF). The results showed that MI-2 significantly inhibited osteoclast formation when used at a concentration of 0.5 μM compared to that in the untreated group. Notably, the effects of MI-2 were concentration-dependent (100 versus 46 ± 15.5, 33 ± 4.0, 3 ± 1.1) (Fig. 1b,c). To quantitatively measure changes in osteoclast formation, the activity of tartrate-resistant acid phosphatase (TRAP), an enzymatic marker of osteoclasts, was analysed following the treatment of monocytes with various concentrations of MI-2 in the presence of RANKL and MCSF. TRAP enzyme activities were gradually reduced following treatment with increasing concentrations of MI-2 (100 versus 87.2 ± 2.1, 83.6 ± 7.1., 37.2 ± 1.2) (Fig. 1d). For negative-control cells (monocytes cultured with MCSF in the absence of RANKL), osteoclast formation was virtually undetectable. In contrast, monocytes cultured in the presence of MCSF and RANKL without MI-2 treatment showed the highest level of osteoclast formation (positive control). We also showed that MALT1 was expressed in monocytes in the presence or absence of RANKL by western blotting (Fig. 1e). MALT1 protein expression was not changed by treatment with RANKL.

### MI-2 reduced osteoclast formation during inflammation

**Figure 2:**
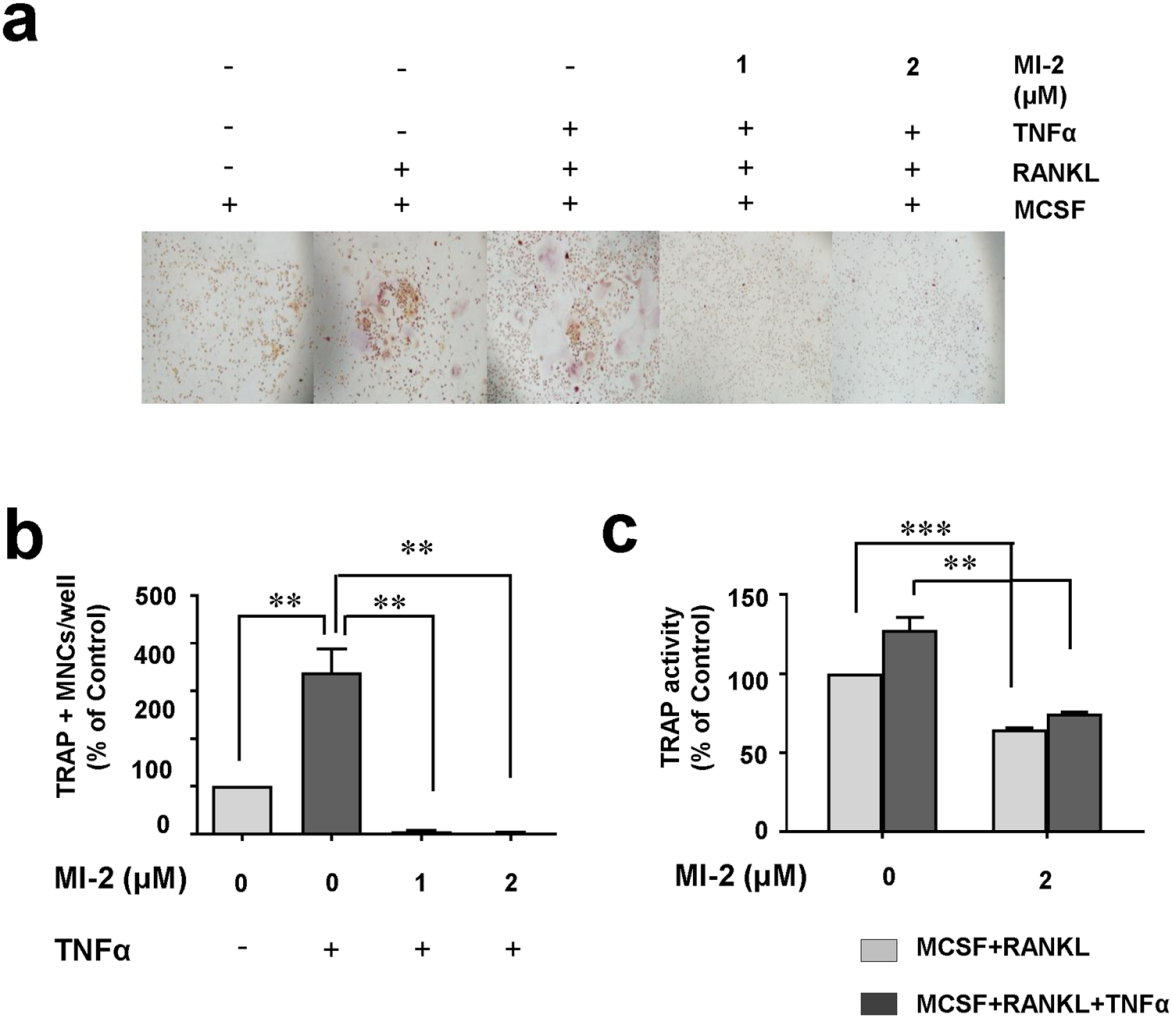
The MALT1 inhibitor MI-2 reduced osteoclast formation in the presence of TNFα. Inhibitory effects of MI-2 on osteoclast formation from monocytes in the presence of 20 ng/mL TNFα, as determined by performing *in vitro* osteoclast-differentiation assays. (**a**) Representative microscopic images from 3 independent experiments are shown. (**b**) Relative abundances (%) of TRAP-positive osteoclasts per well. Pooled results are shown from 3 independent experiments. (**c**) TRAP enzyme activities (%) in untreated cells and cells treated with the indicated concentrations of MI-2. *, **, and *** indicate significant differences from control (untreated) cells based on 2-tailed unpaired Student’s *t*-tests at *P* < 0.05, *P* < 0.01, and *P* < 0.001, respectively. The error bars denote s.e.m. values.

The environment inside the joints of patients with RA is highly inflamed, and large quantities of inflammatory cytokines are produced by various immune cells such as macrophages^13^. Specifically, TNFα was reported to facilitate osteoclast formation in the presence of RANKL^14–16^. Thus, we investigated the inhibitory effects of MI-2 on osteoclast formation in the presence of TNFα. For this, we measured osteoclast formation from human monocytes that were cultured in the presence of MCSF and RANKL, with or without MI-2, and in the presence (339 ± 85.1 versus 5 ± 1.3, 1 ± 2.4, respectively) or absence of TNFα (100 ± 85.1 versus 5 ± 1.3, 1 ± 2.4, respectively). As shown in Fig. 2a, TNFα increased osteoclast formation (100 versus 339 ± 85.1), whereas MI-2 treatment reduced osteoclast formation in the same area in the presence or absence of TNFα (Fig. 2b). Similarly, TRAP activities in MI-2-treated monocytes in the presence (127.7 ± 11.4 versus 74.3 ± 2.0) or absence of TNFα significantly decreased (100 versus 64.5 ± 1.6) (Fig. 2c). Thus, these results suggest that TNFα significantly enhances osteoclast formation from monocytes (Fig. 2a,b) and that MI-2 potently inhibits monocyte differentiation into osteoclasts in the presence and absence of TNFα. These results strongly suggest that MI-2 might strongly inhibit osteoclast formation in inflammatory environments, such as in the inflamed joints of RA patients.

### MI-2 treatment reduced TNFα production by human monocyte-derived macrophages

**Figure 3:**
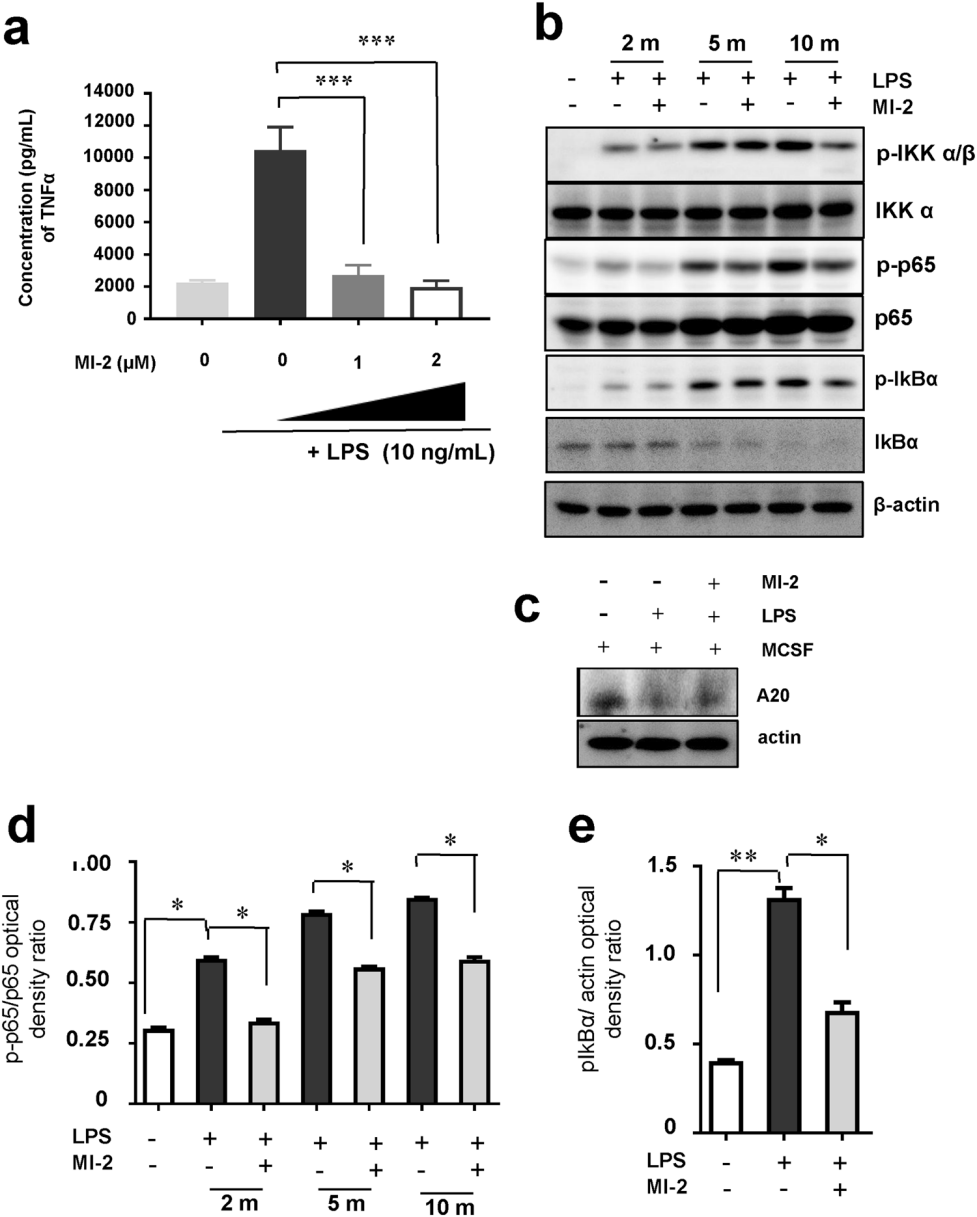
Effects of MI-2 on inflammatory TNFα production in macrophages. (**a**) TNFα concentrations in the culture supernatants from untreated or LPS-treated macrophages, with or without MI-2 treatment, as measured by ELISA. Pooled results are shown from 3 independent experiments. ** and *** indicate significant differences based on 2-tailed unpaired Student’s *t*-tests at *P* < 0.01 and *P* < 0.001, respectively. The error bars show the s.e.m. values. (**b**) Human monocytes were incubated in differentiation medium (α-MEM containing 10% FBS, and 20 ng/mL MCSF), with or without 10 ng/mL LPS, at the indicated time points. Protein expression in the cell lysates was analysed by immunoblotting with antibodies against phosphor-p65 (p-p65), p65, phosphor-IkBα (p-IkBα), and IkBα. β-actin expression was analysed as a loading control. Full-length blots are presented in Supplementary Figure 2. The results shown are representative of 3 independent experiments. (**c**) Human monocytes were incubated in differentiation medium (α-MEM containing 10% FBS, and 20 ng/mL MCSF), with or without 10 ng/mL LPS, for 5 min. Protein expression in the cell lysates was analysed by immunoblotting with antibodies against A20. β-actin expression was analysed as a loading control. Full-length blots are presented in Supplementary Figure 3. The results shown are representative of 3 independent experiments. (**d–e**) The densities of p-p65, p65, p-IkBα, and IkBα bands were measured and normalised to β-actin levels. The p-p65/p65 and p-IkBα/β-actin ratios are shown. Pooled results are shown from 3 independent experiments. * and ** indicate significant differences from control (untreated) cells based on 2-tailed unpaired Student’s *t*-tests at *P* < 0.05 and *P* < 0.01, respectively. The error bars show the s.e.m. values.

The inflammatory cytokine TNFα is critical for facilitating osteoclast formation, as described above. Macrophages are a major source of TNFα and are known to exacerbate RA diseases^17^. Thus, we evaluated whether MI-2 treatment could ameliorate inflammatory responses by reducing the production of the macrophage mediated-inflammatory cytokine TNFα. Accordingly, macrophages were generated from human monocytes that were isolated from peripheral blood to investigate this effect. For this purpose, human recombinant MCSF was used to differentiate monocytes into macrophages. On day 6 after initiating monocyte differentiation, the presence of macrophages was confirmed by analysing the human macrophage marker CD68 by flow cytometry. Most cells expressed CD68, indicating that macrophages were generated (data not shown). TNFα production by macrophages was also evaluated by ELISA following treatment with lipopolysaccharide (LPS) (Fig. 3a). TNFα production by LPS-stimulated macrophages significantly decreased after treatment with MI-2 in a dose-dependent manner (2,233 ± 154 versus 10,468 ± 1,437, 2,702 ± 625, 1,976 ± 395, respectively) (Fig. 3a). To elucidate the mechanism of MI-2-dependent inhibition of TNFα production from macrophages at the molecular level in monocytes, we investigated changes in protein expression or phosphorylation associated with TNFα production of macrophage-related signalling pathways after treating monocytes with MI-2 after the stimulation with LPS. Among the proteins investigated, IKKα/β, a positive regulator of NF-κB activation, was phosphorylated by LPS stimulation in monocytes, but MI-2 treatment reduced the phosphorylation level of IKKα/β, while the total protein levels of IKKα were not changed (Fig. 3b). Furthermore, the expression level of A20 was recovered most efficiently by MI-2 treatment in monocytes after LPS stimulation (Fig. 3c). A20 was previously described as a key protein involved in negatively regulating NF-κB activation and as a target protein regulated by MALT1^18^. Here, we found that A20 expression decreased following LPS-stimulation in monocytes, which was reversed by MALT1 inhibition with MI-2 (Fig. 3c). These findings strongly suggest that MI-2 treatment in monocytes stimulated with LPS prohibit NF-κB activation. Thus, we measured the levels of phosphorylated p65 and IkBα proteins, which consist of active NF-κB heterodimers, and found that they were upregulated after LPS treatment in monocytes and downregulated by MI-2 treatment in the presence of LPS (*P* < 0.05; Fig. 3b,d,e). Phosphorylation of the p65 and IkBα proteins was significantly upregulated by LPS but was blocked by MI-2 treatment after 2, 5, and 10 min of LPS treatment. However, total p65 and IkBα proteins levels were unchanged by treatment with MI-2 (*P* < 0.05; Fig. 3b,d). Taken together, our results strongly suggest that the inhibitory effects of MI-2 on TNFα production are mediated by blocking LPS-induced NF-κB signal activation in macrophages.

### MI-2 ameliorated collagen-induced arthritis in an *in vivo* mouse model

**Figure 4:**
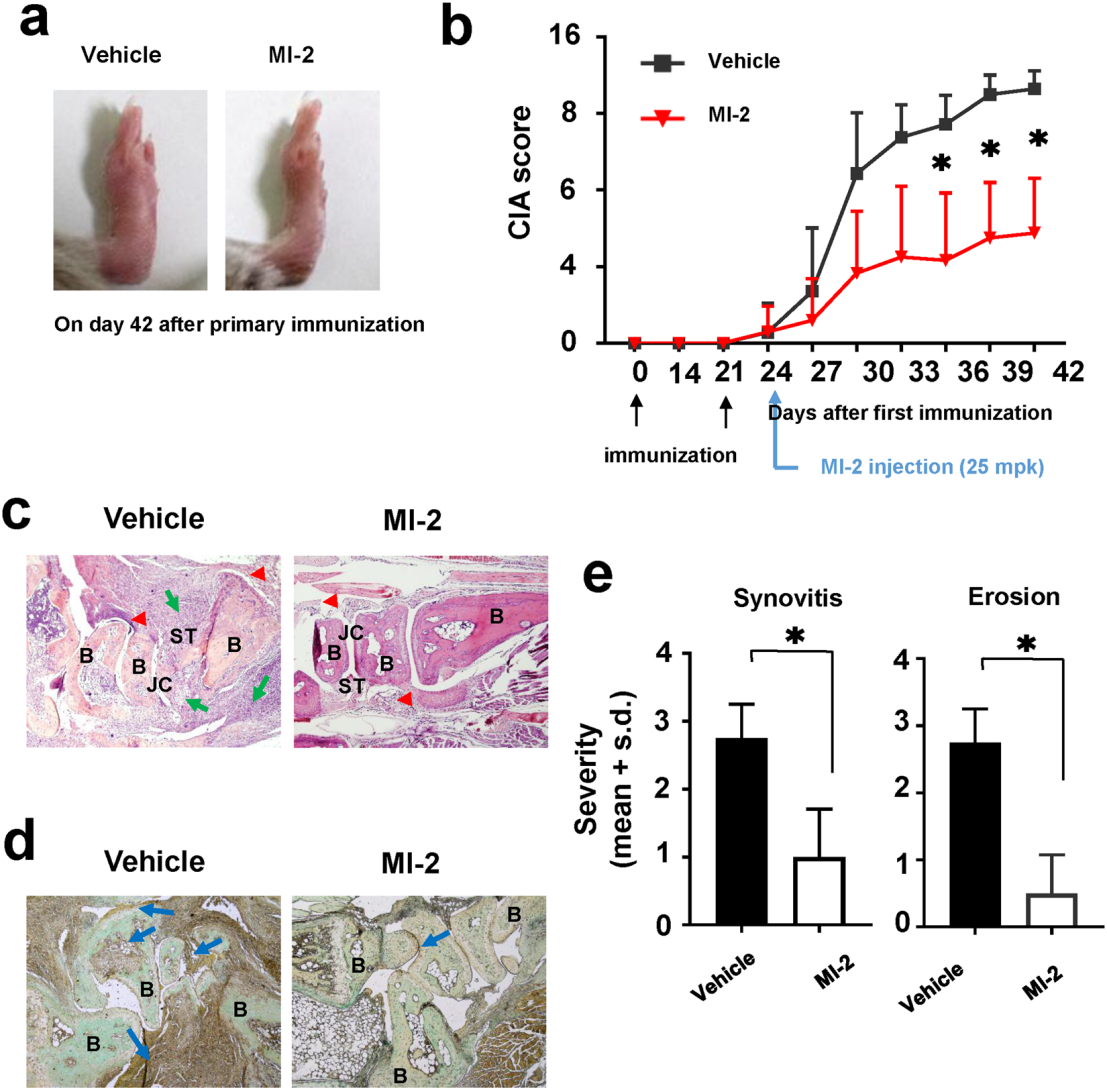
Effects of MI-2 on CIA in a DBA/1 J mouse model. (**a**) Photographs of the paws of CIA mice treated with vehicle or MI-2. (**b**) CIA scores in CIA mice treated with vehicle (n = 6) or MI-2 (n = 6). (**c**) Haematoxylin and eosin-stained sections of a CIA joint 42 d after primary immunisation in CIA mice treated with vehicle or MI-2 by intraperitoneal injection of 25 mpk once per day initiated 24 d after primary immunisation. B: bone, ST: synovial tissue, JC: joint cavity, arrows: sites of inflammation, arrow heads: lining layers of the synovial tissue. (**d**) TRAP-stained sections of a CIA joint 42 d after primary immunisation in CIA mice treated with vehicle or MI-2 by intraperitoneal injection of 25 mpk once per day, initiated 24 d after primary immunisation. B: bone, arrows: TRAP-positive sites, (**e**) Evaluation of synovitis and erosion of bone and cartilage in joint sections in CIA mice treated for 18 d as indicated. *, significant differences based on 2-tailed unpaired Student’s *t*-tests at *P* < 0.05. The error bars show the s.e.m. values.

As described above, 2 potential mechanisms could explain the manner by which MI-2 ameliorates RA, namely, by inhibiting osteoclast formation or by reducing TNFα production. Thus, we tested MI-2 in a mouse model of collagen-induced arthritis (CIA) that shows many clinical and histopathological features of RA. We found that administering MI-2 (25 mg per kg, once per day) for 18 d after the onset of disease symptoms (3 d after the second immunisation with collagen) significantly reduced CIA when compared to those in the vehicle-treated group. We observed that arthritis and paw thickness in CIA mice were ameliorated by MI-2 treatment (Fig. 4a). CIA scores, which is an index of arthritis that is widely used to determine the severity of RA, were measured and compared between the vehicle- and MI-2-treated groups. To eliminate observer bias, we concealed the nature of the treatment (vehicle versus MI-2) from the investigator performing the injections and scored the clinical conditions of the mice. We found that administering MI-2 after the first observation of disease symptoms significantly reduced CIA scores compared to those in the vehicle-treated group (Fig. 4b). Moreover, MI-2 treatment decreased synovitis and the destruction of bone and cartilage (Fig. 4c). In fact, we could observe damaged bones in the paws of vehicle-treated mice, while bones in the paws of the MI-2 treated mice were mostly intact (Fig. 4c). Those destruction of bones in the CIA mice might be caused by an increased production of osteoclasts. Therefore, we compared the TRAP-positive area between the paws of the vehicle-treated mice and the paws of the MI-2 treated mice (Fig. 4d). As shown, a much larger area in the vehicle-treated mice was TRAP-positive compared to the MI-2 treated mice (Fig. 4d). We then scored the histological sections for disease after masking the sample identities. We frequently observed severely inflamed synovial tissues and the destruction of bone and cartilage tissue in the joints of vehicle-treated mice but not in MI-2 treated mice (Synovitis: 2.75 ± 0.5 versus 1.0 ± 0.7; Erosion: 2.75 ± 0.5 versus 1.0 ± 0.5) (Fig. 4e). Because MI-2 treatment was started after we observed the onset of arthritis symptoms, these results strongly suggest that MI-2 could have therapeutic efficacy in treating RA. Moreover, these findings might be dependent at least partially on the inhibitory effects of MI-2 on osteoclast formation, because the severity of bone and cartilage erosion was significantly decreased by MI-2 treatment *in vivo*.

### Mechanism of MI-2-induced inhibition of osteoclast formation

**Figure 5:**
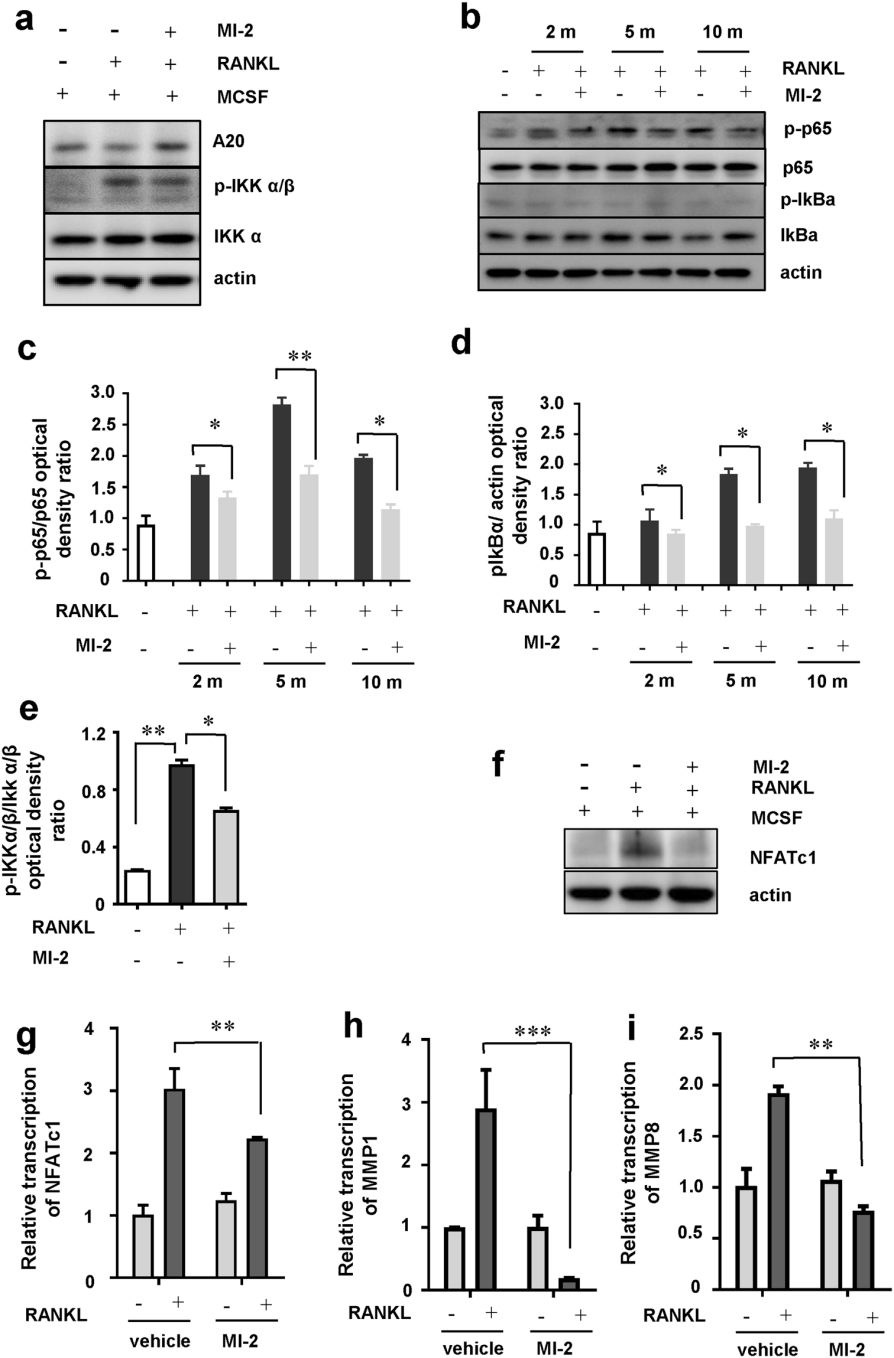
Inhibitory effects of MI-2 on osteoclast formation through blockade of the NF-κB-NFATc1 signalling axis in monocytes. Inhibitory effects of MI-2 on A20 degradation and IKK α/β, p65, and IkBα phosphorylation in monocytes, as determined by *in vitro* osteoclast-differentiation assays. (**a**) Human monocytes were incubated in differentiation medium (α-MEM containing 10% FBS, 20 ng/mL MCSF, and 50 ng/mL RANKL) with or without MI-2. Protein expression in the cell lysates was analysed by immunoblotting with antibodies against pA20, phosphor-IKK α/β, and IKK α. β-actin expression was analysed as a loading control. The results shown are representative of 3 independent experiments. Full-length blots are presented in Supplementary Figure 4; (**b**) Human monocytes were incubated in differentiation medium (α-MEM containing 10% FBS, 20 ng/mL MCSF, and 50 ng/mL RANKL), with or without MI-2, at the indicated time points. Protein expression in the cell lysates was analysed by immunoblotting with antibodies against phosphor-p65 (p-p65), p65, phosphor-IkBα (p-IkBα), and IkBα. β-actin expression was analysed as a loading control. Full-length blots are presented in Supplementary Figure 4. The results shown are representative of 3 independent experiments. (**c–e**) The densities of p-p65, p65, p-IkBα, IkBα, p-Ikkα/β, and Ikkα/β bands were measured and normalised to β-actin levels. The p-p65/p65 and p-IkBα/ β-actin ratios are shown. Pooled results are shown from 3 independent experiments. * and ** indicate significant differences from control (untreated) cells based on 2-tailed unpaired Student’s *t*-tests at *P* < 0.05 and *P* < 0.01, respectively. The error bars show the s.e.m. values. (**f**) Human monocytes were incubated in differentiation medium with or without MI-2. NFATc1 protein expression in the cell lysates was analysed by immunoblotting with antibodies against NFATc1. β-actin expression was analysed as a loading control. Full-length blots are presented in Supplementary Figure 2. The results shown are representative of 3 independent experiments. (**g–i**) Total RNA was extracted from human monocytes incubated in differentiation medium with or without MI-2. NFATc1, MMP1, and MMP8 mRNA levels were measured by qPCR. Pooled results are shown from 3 independent experiments. ** and *** indicate significant differences based on 2-tailed unpaired Student’s *t*-tests at *P* < 0.01 and *P* < 0.001, respectively. The error bars show the s.e.m. values.

As described above, MALT1 is expressed in monocytes, and MI-2-dependent inhibition of osteoclast differentiation is critical for ameliorating RA signs, which suggests that MALT1 might play a critical role in monocyte differentiation into osteoclasts. To elucidate the mechanism of MI-2-dependent inhibition of osteoclast formation at the molecular level in monocytes, we investigated changes in protein expression or phosphorylation associated with osteoclast formation-related signalling pathways after treating monocytes with MI-2 in the presence of MCSF or RANKL. Among the proteins investigated, the expression level of A20 was rescued most efficiently by MI-2 treatment in monocytes after RANKL stimulation (Fig. 5a). A20 has been previously described as a key protein involved in negatively regulating NF-κB activation and as a target protein regulated by MALT1^18^. Here, we found that A20 expression decreased following RANKL-stimulation in monocytes, which was reversed by MALT1 inhibition with MI-2 (Fig. 5a).

Furthermore, IKKα/β, a positive regulator of NF-κB activation, was phosphorylated by RANKL stimulation in monocytes, but MI-2 treatment reduced the phosphorylation level of IKKα/β, while the total protein levels of IKKα/β were not changed (Fig. 5a). These findings strongly suggest that MI-2 treatment in monocytes stimulated with RANKL prohibit NF-κB activation. Thus, we measured the levels of phosphorylated p65 and IkBα proteins, which consist of active NF-κB heterodimers, and found that they were upregulated after RANKL treatment in monocytes and downregulated by MI-2 treatment in the presence of RANKL (Fig. 5b). Phosphorylation of the p65 and IkBα proteins was significantly upregulated by RANKL but was blocked by MI-2 treatment after 10 min of RANKL treatment. However, total p65 and IkBα protein levels were unchanged by treatment with MI-2 (Fig. 5b,c). RANKL-induced osteoclast differentiation required the upregulation of nuclear factor of activated T cells 1 (NFATc1), a key transcriptional factor important for osteoclast formation. Thus, we next investigated whether MI-2-mediated inhibition of phosphorylation of the NF-κB subunits (p65 and IkBα) might affect NFATc1 expression in monocytes. We examined the level of NFATc1 in MI-2-treated or untreated monocytes in the presence of MCSF and RANKL by western blotting. We found that MI-2 potently inhibited the expression and transcription of NFATc1 in monocytes in the presence of MCSF and RANKL based on western blot and qPCR results, respectively (Fig. 5e,f). Thus, these results strongly suggest that RANKL-induced NF-κB activation (phosphorylation of p65 and IkBα proteins) in monocytes is blocked by MI-2-induced blockade of A20 degradation, which results in reduced NFATc1 expression in monocytes in the presence of MCSF and RANKL. Activation of the RANKL/RANK/NF-κB/NFATc1 pathway results in the transcription of osteoclast-characteristic genes that encode proteins such as TRAP, matrix metalloproteinase (MMP) 1, and MMP8^19^. In our study, the transcript levels of *MMP1* and *MMP8* in monocytes were evaluated in the presence of MCSF and RANKL, with or without MI-2 treatment, by qPCR (*MMP1*: 2.90 ± 0.167 versus 0.19 ± 0.004, *P* < 0.005; *MMP8*: 1.92 ± 0.172 versus 0.77 ± 0.044, ±  < 0.01, respectively) (Fig. 5g,h). MI-2-treated monocytes showed reduced *MMP1* and *MMP8* mRNA expression, which is consistent with the observed reduction in osteoclast formation (Fig. 5g,h). The reduced activity of TRAP in MI-2-treated monocytes, compared to that in untreated monocytes, in the presence of MCSF and RANKL had already been confirmed, as shown in Fig. 1d and Fig. 2c.

Thus, our results strongly indicate that the inhibitory effects of MI-2 on osteoclast formation are mediated by blocking RANKL-induced NF-κB/NFATc1 signal activation in monocytes. Taken together, these results strongly suggest that MI-2 treatment might block the NF-κB/NFATc1 axis and decrease osteoclast formation.

## Discussion

MALT1 is a part of the paracaspase pathway and displays proteolytic activity. In addition, MALT1 targets many of the substrates involved in regulating inflammatory responses. Thus, the protease activity of MALT1 has emerged as an interesting therapeutic target^20,21^. To date, most studies on MALT1 have shown that it is a critical modulator of T cell activation. Specifically, MALT1 shows arginine-directed proteolytic activity that is activated after T cell stimulation and cleaves the signalling protein Bcl-10 to mediate T cell receptor-induced cell adhesion to fibronectin^21^. MALT1 also cleaves A20, a protein that inhibits NF-κB, for optimal activation of the transcription factor NF-κB in T cells after T cell antigen receptor-induced signalling^18^. Currently, the known MALT1 protease substrates include TNFAIP3 (A20)^18^, BCL10^21^, CYLD^22^, RELB^22^, MCPIP1^23^, and HOIL1^24^.

In this study, we showed that MALT1 is expressed in monocytes and plays a critical role in regulating TNFα production and osteoclast differentiation. We showed that MALT1 inhibition reduces TNFα production in LPS-stimulated macrophages. We also demonstrated that MALT1-dependant NFATc1 expression is critical for osteoclast differentiation from monocytes. Notably, the NF-κB signalling pathway has been reported to positively regulate NFATc1 expression and TNFα production. As reported previously^24^, LPS activates CD14/Toll-like receptor 4 (TLR4), which results in nuclear translocation of the transcription factor NF-κB and transcriptional activation of many cytokines, including TNFα and other inflammatory molecules. Thus, our results strongly suggest that MALT1 might serve a critical role in NF-κB-mediated inflammatory responses in macrophages. More interestingly, the results showed for the first time that MALT1 inhibition reduces osteoclast formation from monocytes, as mentioned above. We demonstrated that MALT1 inhibition by the small molecule inhibitor MI-2 blocked degradation of the A20 protein, a NF-κB inhibitor protein, resulting in downregulated phosphorylation of Ikkα/β, p65, and IkBα. These are major signalling proteins involved in osteoclast differentiation. Consequently, the reduction of NF-κB activation in monocytes by MI-2 inhibited downstream signalling pathways and specifically reduced transcription of NFATc1, a major transcription factor involved in osteoclast differentiation^25,26^. Moreover, the results of this study indicate that MALT1 inhibition can efficiently block osteoclast formation via TRAP, MMP1, and MMP8 downregulation. Thus, MALT1 inhibition might block NF-κB nuclear translocation, thereby desensitising monocytes to RANKL, which results in reduced NFATc1 expression.

Osteoclast formation and TNFα production from inflammatory macrophages are major aggravating factors of RA. Interestingly, TNFα showed synergistic effects with RANKL in terms of osteoclast formation. Thus, the inflammatory environment might be associated with the facilitation of osteoclast formation in RA joints. In this study, MALT1 inhibition reduced both TNFα production and NF-κB/NFATc1-mediated osteoclast differentiation signalling pathways. Furthermore, the MALT1 inhibitor MI-2 could simultaneously ameliorate RA symptoms, such as bone deformities and inflammation, in a CIA mouse model *in vivo*. These results strongly suggest that MALT1 can potentially serve as an important therapeutic target for RA.

## Materials and Methods

### Human peripheral blood and cell sorting

Peripheral blood from healthy donors was obtained from the Red Cross Blood Center. We followed the guidelines of the Red Cross, and all of the methods and protocols used in this study with human peripheral blood were approved by the Institutional Review Board of the Red Cross. Based on the Red Cross guidelines, informed consent for study participation was obtained and donor information could not be provided. For macrophage-differentiation and osteoclast-formation experiments, human monocytes were isolated from peripheral blood to achieve greater than 95% purity based on negative selection using the RosetteSep Human Monocyte Enrichment Cocktail (StemCell Technologies, Vancouver, BC, Canada). The purity of the isolated monocytes was analysed by flow cytometry using anti-CD14-APC (BioLegend, San Diego, CA, USA), anti-CD16-PE-Cy5 (BioLegend), and anti-CD3-APC-Cy7 (BioLegend) antibodies in phosphate-buffered saline plus 1% foetal bovine serum (FBS) on ice for 10 min. Cells were then washed and analysed. Staining data were collected using a FACSCanto II Cytometer (BD Biosciences). To set the gates for defining positive and negative cells in the multicolour staining, samples were stained with a mixture of all antibodies.

### Mice

DBA/1 J mice were purchased from Dooyeol Biotech (Daejeon, Republic of Korea). We used 8–10-week-old male mice in all experiments. All mice were housed in a pathogen-free animal facility under a 12-h light-dark cycle. All animal experiments were approved by the Institutional Animal Use and Care Committee of the Korea Research Institute of Bioscience and Biotechnology and were performed in accordance with the Guide for the Care and Use of Laboratory Animals published by the US National Institutes of Health.

### Reagents

The MALT1 selective inhibitor, MI-2 (chemical structure shown in Fig. 1a) was purchased from Sigma-Aldrich (St. Louis, MO, USA) and dissolved at a concentration of 30 mM in 100% DMSO as a stock solution, stored at −20 °C, and diluted in medium before each experiment. The final DMSO concentration did not exceed 0.1% throughout this study (all control groups were administered 0.1% DMSO). Antibodies against MALT1, A20, phospho-IκBα, phospho-IKKα/β, IκBα, IKKα, and actin were purchased from Cell Signaling Technology (Danvers, MA, USA). ELISA kits for human TNF were purchased from BioLegend (San Diego, CA, USA). Recombinant human RANKL, MCSF, and TNFα were purchased from Peprotech (Rocky Hill, NJ, USA). LPS was purchased from *In vivo* Gen (San Diego, CA, USA).

### Osteoclast formation and TRAP assays

To analyse osteoclast formation, isolated human monocytes were cultured in a 96-well plate at a density of 1 × 10^5^ cells/well in the presence of human recombinant MCSF (20 ng/mL; Peprotech) in α-MEM (Life Technologies) containing FBS (10%; Life Technologies), l-glutamine (2 mM), and penicillin-streptomycin (Life Technologies) at 37 °C in 5% CO_2_. After 24 h, the cells were incubated in differentiation medium consisting of FBS (10%) with MCSF (50 ng/mL) and RANKL (50 ng/mL) (Peprotech), with or without TNFα (20 ng/mL; Peprotech), and with or without MI-2 at the indicated concentrations for 12 d. On day 12, osteoclast formation was analysed by TRAP staining.

### Generation of monocyte-derived macrophages and TNFα measurement

To promote differentiation into macrophages, isolated human monocytes were cultured in the presence of human recombinant MCSF (20 ng/mL; Peprotech) in RPMI 1640 (Life Technologies, Carlsbad, CA, USA) containing FBS (10%; Life Technologies), l-glutamine (2 mM), and penicillin-streptomycin (Life Technologies) at 37 °C in 5% CO_2_. Human recombinant MCSF was repeatedly added every 2 days after the initiation of the culture. At day 6, generated macrophages were treated with LPS (10 ng/mL) and incubated for 18 h in the presence or absence of various concentrations of MI-2. The culture supernatants were collected and kept at −80 °C until analysis, and TNFα levels in the culture supernatants were measured using the Human TNFα ELISA MAX^TM^ Deluxe Kit following the manufacturer’s procedure (BioLegend). To identify human macrophages, cells were stained with antibodies against human CD68 and TNFα (BioLegend) using the BioLegend Intracellular Staining Kit according to the manufacturer’s instructions prior to analysis by flow cytometry. Staining data were analysed on a MACSQuant®VYB flow cytometer (Miltenyi Biotec, Bergisch Gladbach, Germany).

### Total RNA isolation and real-time reverse transcription polymerase chain reaction (RT-PCR)

Cells were prepared as described above. Total cellular RNA was isolated using the TRIzol reagent (Invitrogen, Carlsbad, CA, USA). Real-time RT-PCR was performed using RNA (20 ng) as the template, qScript cDNA SuperMix for the reverse transcription step, and PerfeCTa qPCR FastMix, UNG, and ROX for PCR (Quanta Biosciences, Gaithersburg, MD, USA). Primer and probe sets (FAM/VIC-labelled) were purchased from Applied Biosystems (Waltham, MA, USA). The results were normalised based on the values obtained for *GAPDH* as detected using TaqMan *GAPDH* control reagents (Applied Biosystems). Real-time qPCR was performed with samples in duplicate using the ABI 7700 Sequence Detection System (Applied Biosystems). For cells from each donor, relative expression levels based on 2^−ΔΔCT^ values are shown as percentages relative to values obtained for the subset with the highest expression.

### Mouse model of collagen-induced arthritis (CIA) to study RA

All DBA/1 (H-2^q^) mice were used at approximately 8–10 weeks of age and had mature immune systems. All mice were immunised intra-dermally at the base of the tail with 200 μg of chicken type II collagen in complete Freund’s adjuvant as described previously. At day 20 after the initial immunisation, a second immunisation with 200 μg of chicken type II collagen in incomplete Freund’s adjuvant was performed.

### CIA scoring and histological assessment of arthritis

Animals were scored for clinical signs of arthritis as follows: 0 = normal; 1 = 1 toe inflamed and swollen; 2 = more than 1 toe inflamed and swollen (but not the entire paw), or mild swelling of the entire paw; 3 = entire paw inflamed and swollen; 4 = very inflamed and swollen or ankylosed paw. Each limb was graded accordingly, with a maximum score of 16 for each mouse. After completion of the experiment, mice were sacrificed and hind paws were immersion-fixed in buffered formalin (10%, v/v) and decalcified with ethylenediaminetetraacetic acid (5.5%) in buffered formalin. For the histological assessments of arthritis, arthritic mice were euthanised up to 18 d after disease onset. Joints were decalcified and paraffin-embedded, and sections (10 μm) were stained (haematoxylin and eosin) using conventional histological methods. Joints were classified according to the presence or absence of inflammatory cell infiltrates (defined as focal accumulation of leukocytes). Histological analysis was performed by investigators blinded to the experimental conditions. These individuals judged the extent of synovitis or bone and/or cartilage destruction on a scale as follows: grade 0 (no signs of inflammation); grade 1 (mild inflammation with hyperplasia of the synovial lining layer, without cartilage destruction) to grades 2 through 4 (increasing degrees of inflammatory cell infiltrate or cartilage and bone destruction).

### Western blotting

Cells were prepared as described above and lysed on ice in buffer (30 mM Tris HCl, pH 8.0; 75 mM NaCl; 10% glycerol; and 1% Triton X-100) containing proteinase inhibitor cocktail (Cell Signaling Technology) at a 1: 100 dilution. Cellular lysates were centrifuged at 12,000 × *g* for 10 min at 4 °C, and supernatants were collected after centrifugation. Protein contents were quantified using the Micro BCA Protein Assay (Pierce) according to the manufacturer’s guidelines with bovine serum albumin as a standard. Samples were prepared for sodium dodecyl sulphate-polyacrylamide gel electrophoresis (SDS-PAGE) by boiling at 100 °C in 2× Laemmli Sample Buffer (Bio-Rad) containing β-mercaptoethanol (5%). Cellular proteins (40 µg) were separated by SDS-PAGE at 100 V using the PageRuler Plus Prestained Protein Ladder (Thermo Scientific) as the marker. After electrophoresis, proteins were transferred to an Immun-Blot polyvinylidene difluoride membrane (Bio-Rad) for 1 h at room temperature (RT) using a Mini Trans-Blot Cell (Bio-Rad). Following transfer, the membrane was washed in Tris-buffered saline (20 mM Tris, 136 mM NaCl; pH 7.4) with 0.1% Tween 20 (TBST), blocked for 1 h in TBST (with 5% non-fat dried milk), incubated overnight at 4 °C in the same solution containing appropriate dilutions of antibodies against human MALT1, A20, p-IKK α/β, IKK α/β, p-p65, p65, p-IKB α, IKB α, and actin (Cell Signaling Technology), washed with TBST, incubated at RT with a 1:10,000-dilution of a horseradish peroxidase-conjugated goat anti-mouse antibody or anti-rabbit antibody (Cell Signaling Technology) in TBST (with 5% non-fat dried milk) for 1 h, and then washed with TBST at RT. Protein bands were visualised using the SuperSignal West Pico Chemiluminescent Substrate (Pierce).

### Statistical analysis

Analysis of variance was used for multiple comparisons. Unpaired-*t* tests were performed to analyse differences between 2 groups. Statistical analyses were performed using the Prism software package (GraphPad Software, San Diego, CA, USA). Differences with *P* values of 0.05 or less were considered significant.

## Electronic supplementary material

Supplementary Information
