# Supplementary material for: Mucosa-associated lymphoid tissue lymphoma translocation 1 as a novel therapeutic target for rheumatoid arthritis
Source: Sci Rep. 2017 Sep 19;7:11889. doi: 10.1038/s41598-017-12349-9 (PMC5605699; doi:10.1038/s41598-017-12349-9)
Supplement: Supplementary file 1 — Supplementary Information [file 41598_2017_12349_MOESM1_ESM.pdf]

Supplementary Information

Mucosa-associated lymphoid tissue lymphoma translocation 1 as a novel therapeutic target for rheumatoid arthritis

Chang Hoon Lee*, Su Jeong Bae and Miok Kim

Supplementary figure 1: Immunoblot analysis of MALT-1 in human monocytes.

a.

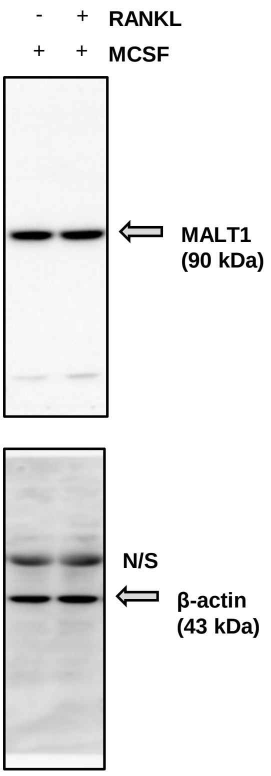

(a) Human monocytes were used to assesses the expression of MALT1 using immunoblot method with a use of β –actin as a loading control. Human monocytes were incubated in differentiation medium (20 ng/mL M-CSF and 50 ng/mL RANKL in α-MEM containing 10% FBS). The level of β-actin was analysed as a loading control. N/S indicates non-specific band.

Supplementary figure 2: Immunoblot analysis of p-IKK α/β, IKKα, p-IkBα, IkBα, p-p65 and p65 in human monocytes.

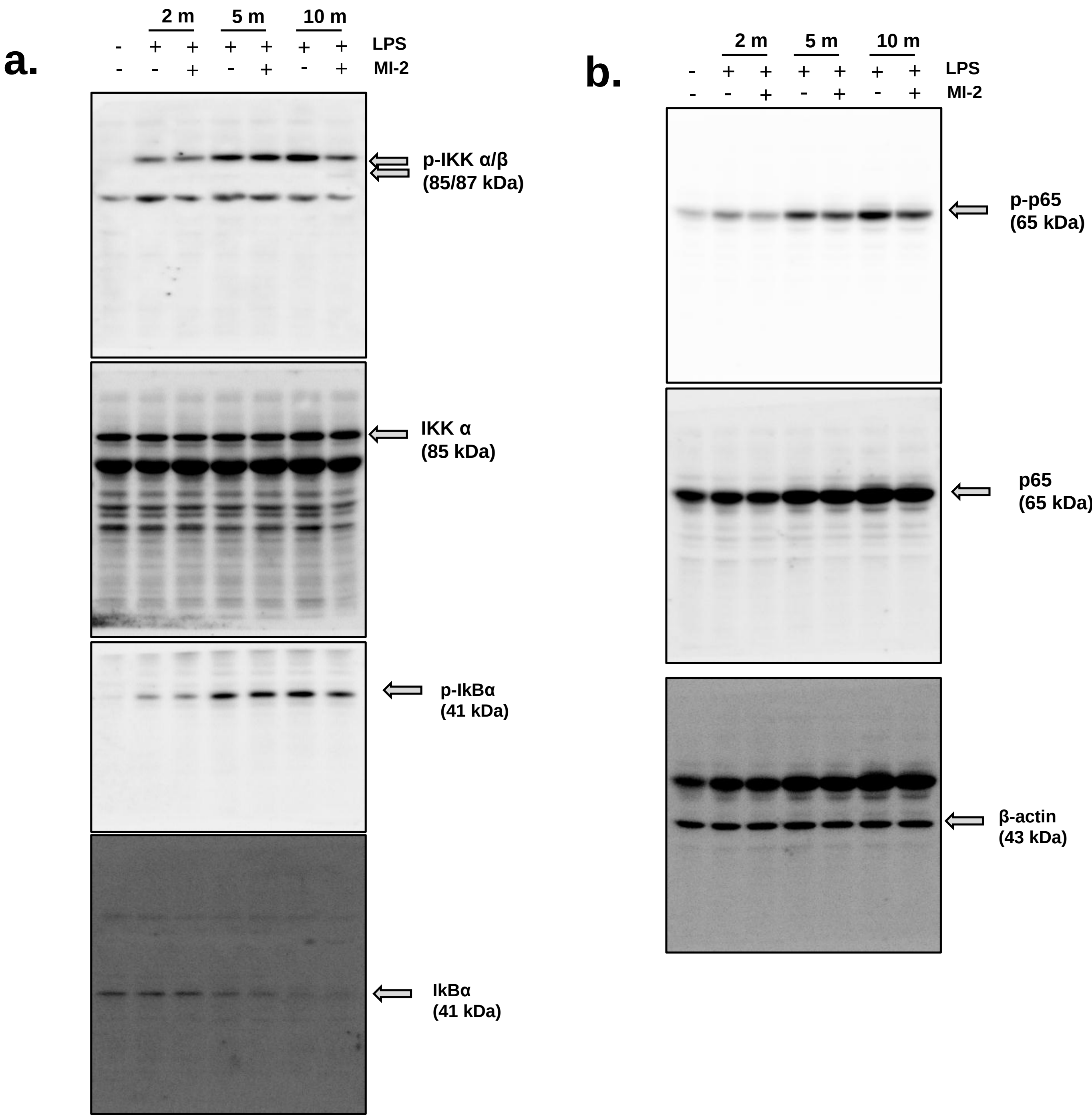

(a) Human monocytes were incubated in differentiation medium (20 ng/mL M-CSF and 10 ng/mL LPS in α-MEM containing 10% FBS) in the presence or absence of MI-2. Human monocytes were used to assesses the expression of p-IKK α/β, IKKα, p-IkBα and IkBα using immunoblot method with a use of β –actin as a loading control. (b) Protein expression in the cell lysates was analysed by immunoblot analysis with antibodies p-p65 and p65 against. The level of β-actin was analysed as a loading control. N/S indicates non-specific band.

Supplementary figure 3: Immunoblot analysis of A20 in human monocytes.

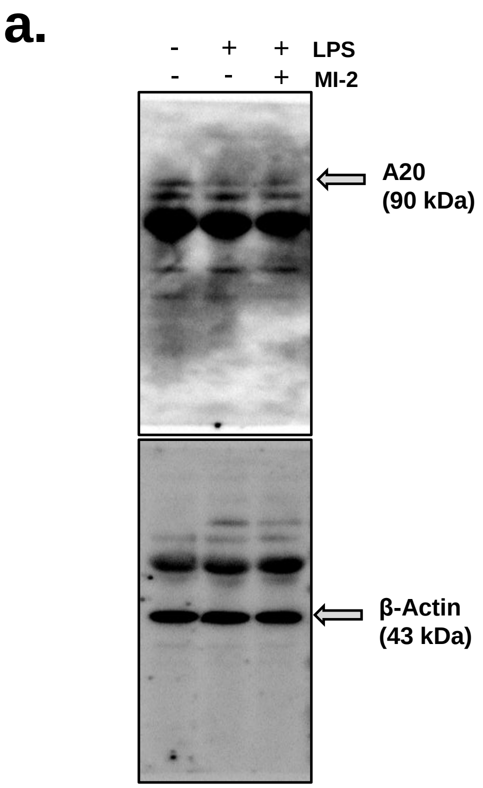

(a) Human monocytes were incubated in differentiation medium (20 ng/mL M-CSF and 10 ng/mL LPS in α-MEM containing 10% FBS) in the presence or absence of MI-2. Human monocytes were used to assesses the expression of A20 using immunoblot method with a use of β –actin as a loading control. The level of β-actin was analysed as a loading control. N/S indicates non-specific band.

Supplementary figure 4: Immunoblot analysis of MALT1, A20, and p-IKK α/β in human monocytes.

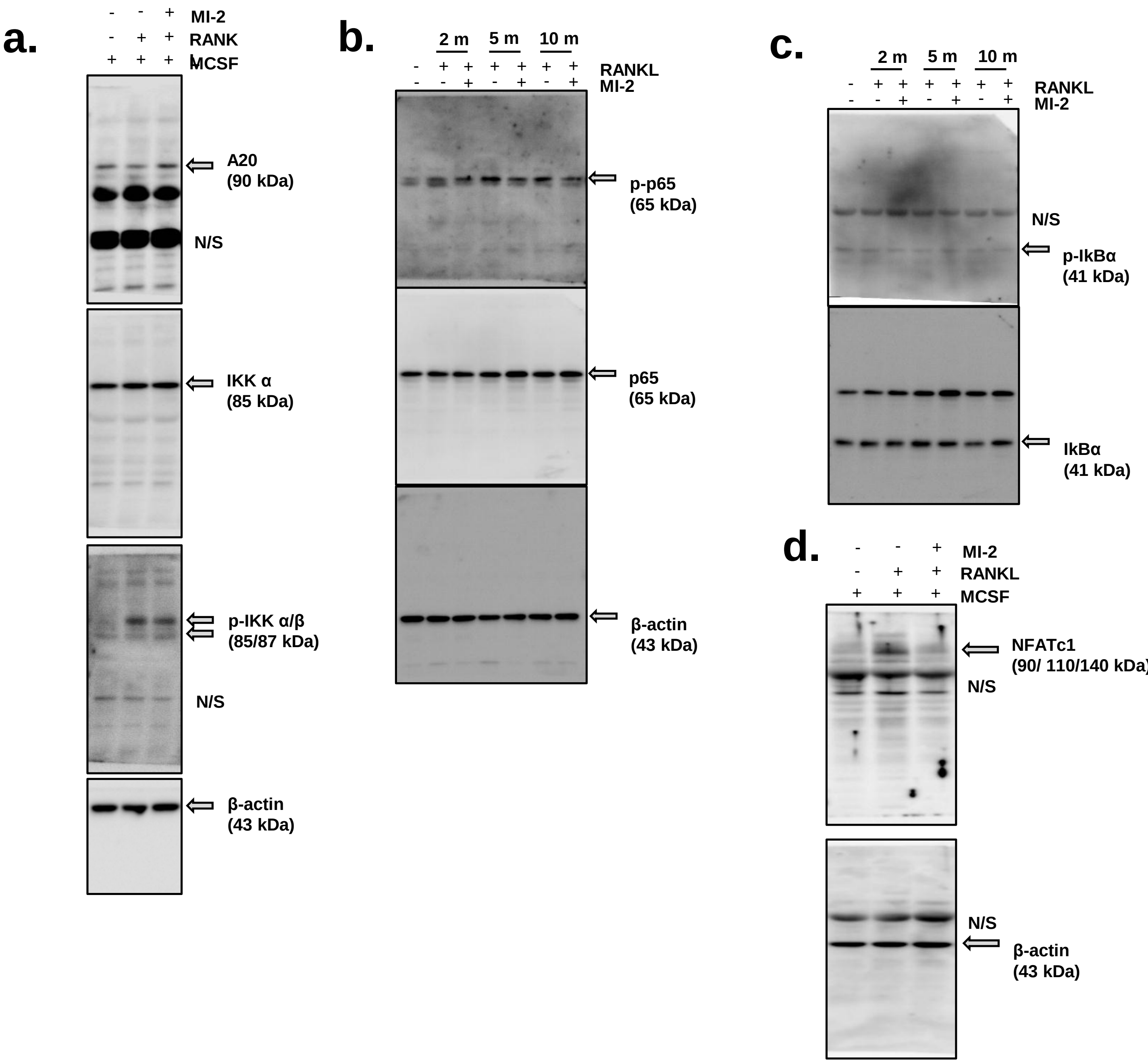

(a) Human monocytes were incubated in differentiation medium (20 ng/mL M-CSF and 50 ng/mL RANKL in α-MEM containing 10% FBS) in the presence or absence of MI-2. Protein expression in the cell lysates was analysed by immunoblot analysis with antibodies against p A20 and phosphor-IKK α/β and IKK α. (b) Human monocytes were incubated with stimulation of 50 ng/mL RANKL in the presence or absence of MI-2 for indicated time durations. Cell lysates from human monocytes were used to assesses the level of phosphor-p65 and expression of p65 and (c) to assesses the level of phosphor-IkBα and expression of IkBα using immunoblot method with a use of β–actin as a loading control. (d) Protein expression in the cell lysates was analysed by immunoblot analysis with antibodies against p NFATc1. The level of β-actin was analysed as a loading control. N/S indicates non-specific band.
